# The effects of body position and actual execution on motor imagery of locomotor tasks in people with a lower-limb amputation

**DOI:** 10.1038/s41598-021-93240-6

**Published:** 2021-07-02

**Authors:** Arnaud Saimpont, Francine Malouin, Anne Durand, Catherine Mercier, Franck di Rienzo, Elodie Saruco, Christian Collet, Aymeric Guillot, Philip L. Jackson

**Affiliations:** 1 Laboratoire Interuniversitaire de Biologie de la Motricité (LIBM) EA 7424, Université Claude Bernard Lyon 1, Univ Lyon, F-69622 Villeurbanne Cedex, France; 2 Department of Rehabilitation, Université Laval, Quebec City, Canada; 3 Centre Interdisciplinaire de Recherche en Réadaptation et Intégration Sociale (Cirris), Quebec City, Canada; 4 Centre intégré universitaire de santé et de services sociaux (CIUSSS) de la Capitale-Nationale, Quebec City, Canada; 5 Department of Neurology, BG University Clinic Bergmannsheil, Ruhr-University Bochum, Bochum, Germany; 6 CERVO Brain Research Center, Quebec City, Canada; 7 School of Psychology, Université Laval, Quebec City, Canada

**Keywords:** Neuroscience, Psychology, Neurology

## Abstract

Motor imagery (MI) is usually facilitated when performed in a congruent body position to the imagined movement, as well as after actual execution (AE). A lower-limb amputation (LLA) results in important structural and functional changes in the sensorimotor system, which can alter MI. In this study, we investigated the effects of body position and AE on the temporal characteristics of MI in people with LLA. Ten participants with LLA (mean age = 59.6 ± 13.9 years, four females) and ten gender- and age-matched healthy control participants (mean age = 60.1 ± 15.4 years, four females) were included. They performed two locomotor-related tasks (a walking task and the Timed Up and Go task) while MI times were measured in different conditions (in congruent/incongruent positions and before/after AE). We showed that MI times were significantly shorter when participants imagined walking in a congruent-standing position compared to an incongruent-sitting position, and when performing MI after actual walking compared to before, in both groups. Shorter MI times in the congruent position and after AE suggest an improvement of MI’s temporal accuracy (i.e. the ability to match AE time during MI) in healthy individuals but not in the LLA group.

## Introduction

Approximately 30 years of research have revealed many similarities between imagined and executed movements at both the behavioural (temporal) and neurophysiological (physiological responses and brain activity) levels^1–6^. In parallel with these studies, the usefulness of motor imagery (MI) training, namely the repeated practice of MI to improve motor learning and functional rehabilitation, has been investigated and promoted^7–12^.

Only a few studies have tried to disentangle whether changes in MI ability occur after a lower-limb amputation (LLA) despite the critical structural and functional alterations occurring in the sensorimotor system after LLA and the potential for MI training in this population^13,14^. The findings so far indicate that MI vividness (i.e. the clarity of images and intensity of sensations of movements during MI) is reduced for movements involving the distal segment of the amputated lower limb but preserved for the proximal segment^15,16^. Furthermore, the time since walking with a prosthesis is positively correlated with the vividness scores for the amputated side^15^. The temporal accuracy of MI, namely the ability to match actual execution (AE) time during MI, is preserved in chronic LLA for simple lower-limb movements (hip abduction) that can be physically executed^15^. In contrast, at the beginning of locomotor prosthetic training in the subacute stage of LLA, MI times were found to be shorter than AE times for locomotor-related tasks^16^. Interestingly, this temporal discrepancy subsided with prosthesis training, indicating a better estimate of AE times during MI with more motor experience. Hence, both MI vividness and MI’s temporal accuracy improve with practice in people with LLA.

Computational approaches of motor control may help to interpret the impact of motor experience on MI^17,18^. These approaches postulate the existence of internal models that would generate motor commands to achieve the goal of a movement (inverse model) and, in parallel, relate motor commands with their sensory consequences (forward model). These models allow the feedforward control of movements—a predictive mode of control that does not require sensory feedback. However, during MI, feedback is typically not available. Motor imagery would thus reproduce the predictive operations of the internal models^19,20^. When one actually executes a movement, one compares motor predictions with the actual achievement of the goal and the sensory feedback, and one uses any difference to refine or update the models^17^. Hence, after locomotor prosthetic training, people with LLA may have refined their internal models (altered by the amputation) in long-term procedural memory through many trials and errors, which, in turn, may have enhanced the accuracy of MI.

Other factors, such as body position during MI and the movement being executed or not before MI, can influence MI in healthy adults. First, MI of upper-limb movements appears to be facilitated (i.e. shorter MI times) when one’s body position is congruent with the position at the beginning of the movement to imagine^21–30^. Furthermore, in younger and older adults, Saimpont et al.^31^ showed that imagined walking times are shorter when MI is performed in a congruent position (i.e. standing) rather than in an incongruent one (i.e. sitting), revealing that the MI of locomotor-related movements may also be influenced by body position. These results also fit with the theory of internal models, which are thought to take into account the ongoing flow of sensory information from the body segments to generate their predictions^17^. Alternatively, the decrease in MI times in congruent positions may result from a facilitation of information processing, with easier or faster access to the sensory information needed to create and manipulate mental images and body sensations. Second, another way to influence the temporal characteristics of MI in healthy individuals is to physically perform the task just before MI^19,32^; see also^10,11^. For example, the variability of MI times in a walking task significantly decreases when MI trials alternate with AE trials compared to when they are all performed before AE^32^. These results are also coherent with the theory of internal models. Similarly to the impact of motor experience, prior AE may help to refine the motor predictions used for MI by improving their accuracy, although in short-term memory in this case.

In sum, motor experience, actual body position and prior AE influence MI. Whether the effects of body position and AE would also be observed after LLA has yet not been tested. This study assesses whether these factors modulate the time to imagine two locomotor-related tasks, a walking task and timed-up-and-go (TUG) tasks, both in people with LLA and healthy individuals. Specifically, we anticipated shorter MI times when healthy adults adopted a congruent body position and when MI followed AE in. In the LLA group, we expected that MI times would increase under these conditions provided that individuals underestimated AE times during the MI of locomotor tasks^16^ and that a refinement of motor predictions fruent body position and after AE would lead to a better estimate of AE times. We thus expected different results regarding MI’s temporal accuracy in people with LLA and healthy individuals—namely, we anticipated an underestimation and an overestimation of AE times during MI in the LLA and control (CTL) groups, respectively^16,31^.

## Results

Table 1 summarizes the characteristics of the participants in the LLA and CTL groups.

**Table 1:** Characteristics of the participants in the LLA and CTL groups.

	LLA											CTL			
Subject	Age (years)	Gender	Mean KVIQ score (/5)	Etiology of Amputation	Amputation level	Time since amputation (weeks)	Walking time with pneumatic prosthesis (min)	Time since prosthesis wearing (days)	Mean prosthesis wearing time per day* (hours)	Phantom limb pain** (/100)	Stump pain** (/100)	Subject	Age (years)	Gender	Mean KVIQ score (/5)
LLA 1	40	M	3.1	Tr	TT	19	55	15	5	5	0	CTL 1	38	M	3.4
LLA 2	53	F	2.1	V	TF	20	220	38	4	0	0	CTL 2	53	F	3.8
LLA 3	73	M	1.3	V	TT	47	295	34	3	0	5	CTL 3	75	M	3.1
LLA 4	67	F	3.3	Tr	TF	28	390	47	5	0	0	CTL 4	69	F	4.9
LLA 5	48	M	2.2	Tr	TF	31	167	55	13	0	0	CTL 5	53	M	2.4
LLA 6	57	M	2.5	Tr	TT	25	219	21	5	20	0	CTL 6	58	M	2.8
LLA 7	53	F	3.6	Tr	TT	27	35	8	4	0	30	CTL 7	54	F	3.1
LLA 8	44	F	2.8	Tu	TT	33	0	8	4	30	0	CTL 8	41	F	4.3
LLA 9	78	M	1.9	I	TT	26	382	23	4	0	0	CTL 9	80	M	4.4
LLA 10	78	M	3.3	V	TT	22	457	23	3	10	0	CTL 10	81	M	4.7
**Mean (SD)**	**59.6** (13.9)		**2.58** (0.72)			**27.8** (8.1)	**222** (160)	**27.2** (15.9)	**5.00** (2.91)	**6.5** (10.6)	**3.5** (9.4)		**60.1** (15.4)		**3.70** (0.86)

M, Male; F, Female; I, Infectious; Tr, Traumatic; Tu, Tumoral; V, Vascular; TT, Transtibial; TF, Transfemoral.

*During the week before testing, **At the beginning of testing.

### Actual execution times

Table 2 reports mean AE times in the two groups for the two tasks. As expected, results showed a significant *Group* effect in both the walking task (*F*_1,16_ = 36.67, *p* < 0.001, η_p_^2^ = 0.70, post hoc power = 1.00) and the TUG task (*F*_1,16_ = 34.77, *p* < 0.001, η_p_^2^ = 0.69, post hoc power = 1.00), with the LLA group taking more time to actually perform the walking and TUG tasks than the CTL group. There was no significant *Position* effect (walking task: *p* = 0.161, *B*_HN(0, 4.75)_ = 0.35) ; TUG task: *p* = 0.916, *B*_HN(0, 9.59)_ = 0.02) and *Position* × *Group* interaction (walking task: *p* = 0.210, *B*_HN(0, 4.75)_ = 0.40; TUG task: *p* = 0.898, *B*_HN(0, 9.59)_ = 0.04), thus showing stable physical performance (AE times) between conditions throughout testing in both groups.

**Table 2:** Mean (± SD) AE times (seconds) in the LLA (n = 8) and CTL (n = 10) groups for the walking task, in the blocks where MI was performed in the standing-congruent and supine-incongruent positions; and for the TUG task, in the blocks where MI was performed in the sitting-congruent and supine-incongruent positions.

Group	Walking task		TUG task	
	Standing	Supine	Sitting	Supine
LLA	13.1 ± 3.5	12.5 ± 3.1	26.0 ± 6.3	26.1 ± 6.7
CTL	6.6 ± 0.7	6.5 ± 0.7	13.7 ± 1.6	13.6 ± 1.9

### Motor imagery times

**Figure 1:**
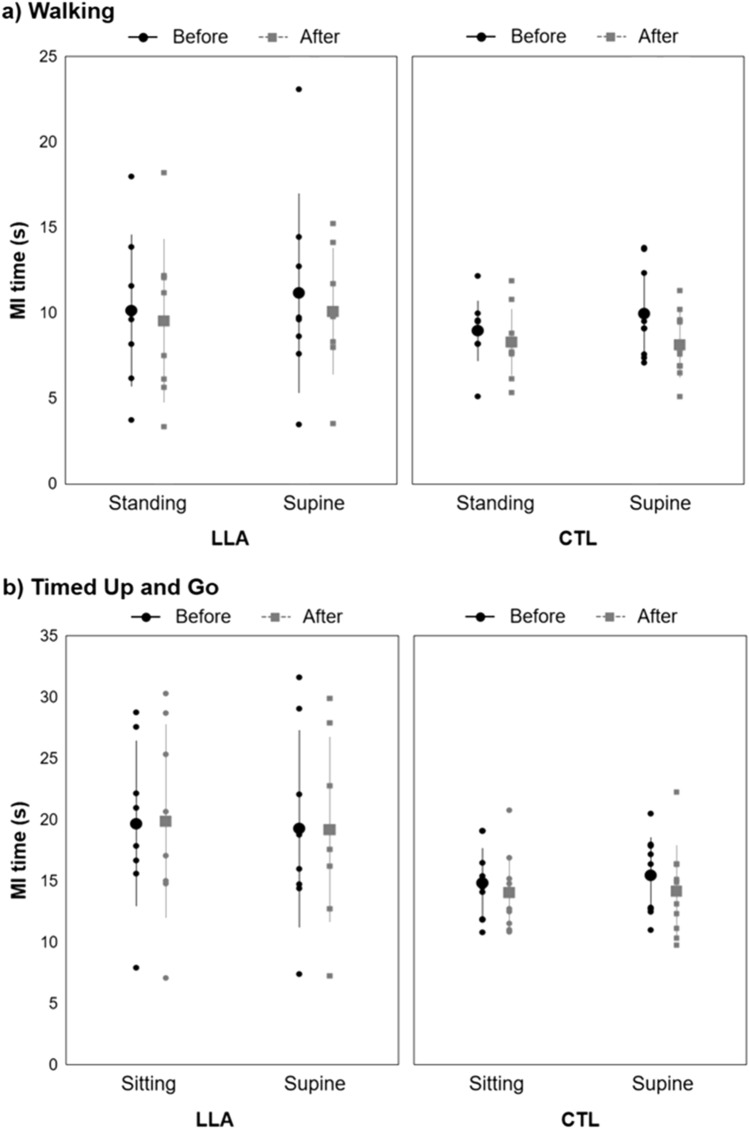
Mean (± SD) MI times (in seconds) in the LLA (n = 8) and CTL (n = 10) groups for (**a**) the walking task, in the standing and supine positions, before and after AE, and (**b**) the TUG task, in the sitting and supine positions, before and after AE. Small dots and squares represent individual data, large points and squares, mean data, and bars, SD. In the walking task, there was a significant *Position* effect (*p* = .030) with shorter MI times in the standing-congruent position than in the supine-incongruent position. There was also a significant *Order* effect (*p* = .009) with shorter MI times after AE than before AE. In the TUG task, MI times tended (*p* = .080) to be longer in the LLA group than in the CTL group.

Figure 1 illustrates MI times in the two groups for the two tasks. In the walking task, results showed a significant *Position* effect (*F*_1,16_ = 5.63, *p* = 0.030, η_p_^2^ = 0.26, post hoc power = 0.61), with shorter MI times in the standing-congruent position (mean = 9.2 s ± 3.3) than in the supine-incongruent position (mean = 9.7 s ± 3.7). There was also a significant *Order* effect (*F*_1,16_ = 8.80, *p* = 0.009, η_p_^2^ = 0.36, post hoc power = 0.80) as MI times were shorter after AE (mean = 8.9 s ± 3.2) than before AE (mean = 9.7 s ± 3.7). The *Group* effect did not reach significance (*p* = 0.377, *B*_HN(0, 5.12)_ = 1.05) nor did the different interactions: *Position* × *Group* (*p* = 0.450, *B*_HN(0, 4.86)_ = 0.24), *Order* × *Group* (*p* = 0.447, *B*_HN(0, 4.99)_ = 0.29) and *Position* × *Order* (*p* = 0.239, *B*_HN(0, 4.99)_ = 0.49) and *Position* × *Order* × *Group* (*p* = 0.655, *B*_HN(0, 4.99)_ = 0.48).

In the TUG task, there was a trend for a *Group* effect (*F*_1,16_ = 3.61, *p* = 0.080, η_p_^2^ = 0.18, post hoc power = 0.43), with MI times tending to be longer in the LLA group (mean = 19.5 s ± 7.3) than in the CTL group (mean = 14.6 s ± 3.1). No other effects or interactions reached significance: *Position* (*p* = 0.820, *B*_HN(0, 8.41)_ = 0.03), *Order* (*p* = 0.311, *B*_HN(0, 8.53)_ = 0.19), *Position* × *Group* (*p* = 0.236, *B*_HN(0, 8.41)_ = 0.30), *Order* × *Group* (*p* = 0.262, *B*_HN(0, 8.53)_ = 0.35) and *Position* × *Order* (*p* = 0.309, *B*_HN(0, 8.53)_ = 0.17) and *Position* × *Order* × *Group* (*p* = 0.690, *B*_HN(0, 8.53)_ = 0.09).

In the walking task, mean vividness scores were 2.8 ± 0.8 in the LLA group and 3.0 ± 0.9 in the CTL group. In the TUG task, these were 2.9 ± 0.6 in the LLA group and 2.9 ± 1.1 in the CTL group. In one modality of MI at least, all participants reported a score ≥ 2 in both tasks, indicating an acceptable compliance level during MI.

### Temporal accuracy of MI

#### Group-specificity

**Figure 2:**
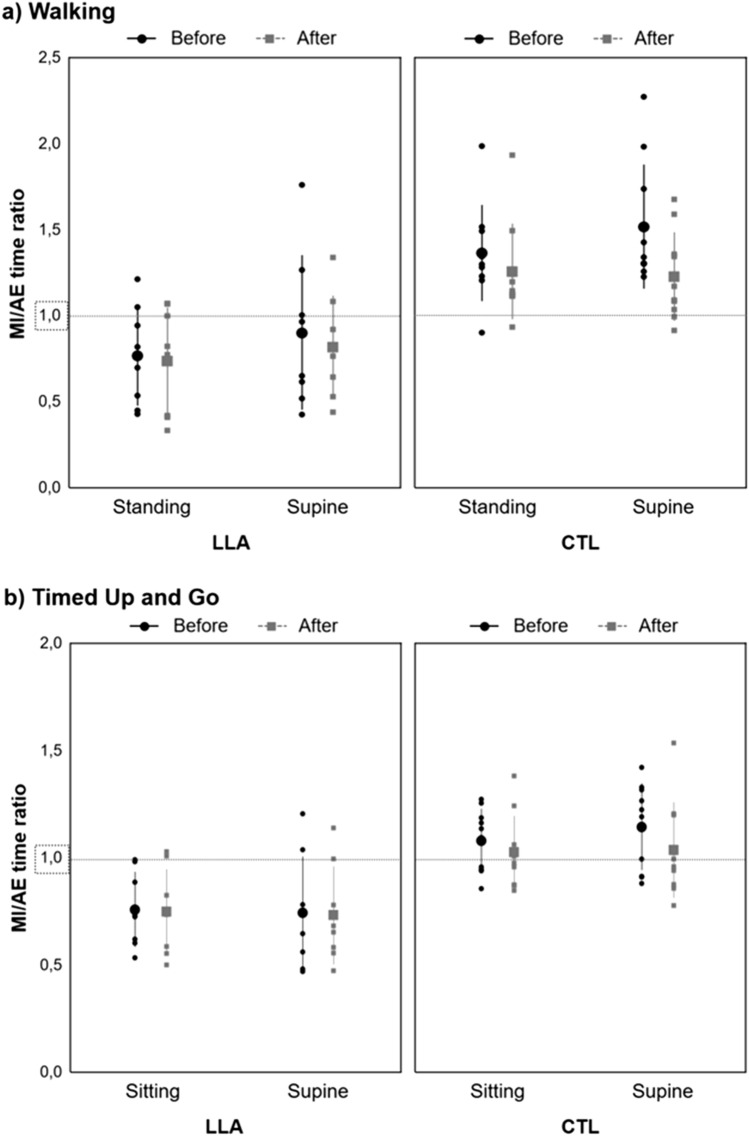
Mean (± SD) MI/AE time ratios in the LLA (n = 8) and CTL (n = 10) groups for (**a**) the walking task, in the standing and supine positions, before and after AE, and (**b**) the TUG task, in the sitting and supine positions, before and after AE. Time ratios under 1 (dot lines) indicate that MI times were shorter than AE times, and conversely for MI/AE time ratios above 1. Small dots and squares represent individual data, large points and squares, mean data, and bars, SD. In the walking task, there was a significant *Group* effect (*p* < .001) with lower ratios in the LLA group compared to the CTL group, a significant *Position* effect (*p* = .030) with lower ratios in the standing-congruent position than in the supine-incongruent position. There was also a significant *Order* effect (*p* = .003) with lower ratios after AE than before AE. In the TUG task, there was a significant *Group* effect (*p* = .003) with lower ratios in the LLA group compared to the CTL group. In both tasks, note that the LLA group underestimated AE times during MI while the CTL group overestimated them.

Figure 2 shows MI/AE time ratios in the two groups for the two tasks. In the walking task, the analysis showed a significant *Group* effect (*F*_1,16_ = 30.02, *p* < 0.001, η_p_^2^ = 0.65, post hoc power = 1.00), with lower MI/AE time ratios in the LLA group (mean = 0.80 ± 0.34) compared to the CTL group (mean = 1.34 ± 0.30). Results also revealed a significant *Position* effect (*F*_1,16_ = 5.70, *p* = 0.030, η_p_^2^ = 0.26, post hoc power = 0.61), MI/AE time ratios being lower in the standing-congruent position (mean = 1.06 ± 0.40) than in the supine-incongruent position (mean = 1.14 ± 0.44). There was also a significant *Order* effect (*F*_1,16_ = 12.41, *p* = 0.003, η_p_^2^ = 0.44, post hoc power = 0.91), with lower MI/AE time ratios after AE (mean = 1.03 ± 0.36) than before AE (mean = 1.17 ± 0.46). No interactions were significant: *Position* × *Group* (*p* = 0.949, *B*_HN(0, 0.57)_ = 0.22), *Order* × *Group* (*p* = 0.742, *B*_HN(0, 0.59)_ = 0.69), *Position* × *Order* (*p* = 0.632, *B*_HN(0, 0.59)_ = 0.76) and *Position* × *Order* × *Group* (*p* = 0.375, *B*_HN(0, 0.59)_ = 0.90). In the TUG task, results showed a significant *Group* effect (*F*_1,16_ = 12.66, *p* = 0.003, η_p_^2^ = 0.44, post hoc power = 0.92), with lower MI/AE time ratios in the LLA group (mean = 0.75 ± 0.22) compared to the CTL group (mean = 1.07 ± 0.19). No other effects or interactions were significant: *Position* (*p* = 0.633, *B*_HN(0, 0.47)_ = 0.73), *Order* (*p* = 0.100, *B*_HN(0, 0.48)_ = 0.97), *Position* × *Group* (*p* = 0.257, *B*_HN(0, 0.47)_ = 0.94), *Order* × *Group* (*p* = 0.136, *B*_HN(0, 0.48)_ = 0.97) and *Position* × *Order* (*p* = 0.281, *B*_HN(0, 0.48)_ = 0.93) and *Position* × *Order* × *Group* (*p* = 0.403, *B*_HN(0, 0.48)_ = 0.89).

#### Task-consistency

**Figure 3:**
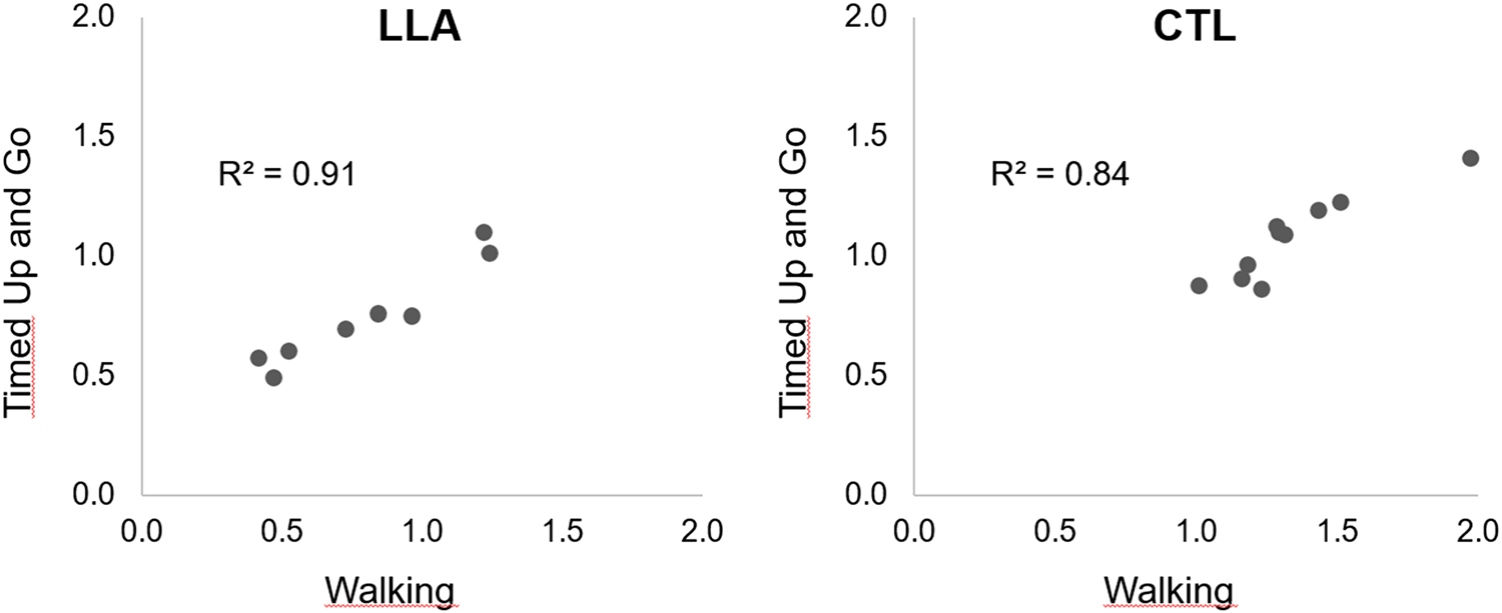
Relationship between the TUG and Walking MI/AE time ratios (each point represents the mean of all conditions for a given participant) in the LLA and CTL groups. Spearman’s Rho were statistically significant in both groups.

Figure 3 shows the relation between the TUG and Walking MI/AE time ratios in the two groups. The Spearman tests revealed that the MI/AE time ratios were significantly correlated for the two tasks in both the LLA (Rho = 0.83; *p* < 0.001) and CTL (Rho = 0.76; *p* < 0.001) groups. Note also that, consistent with the mean data, the majority of participants underestimated AE times during MI in the LLA group, while the majority overestimated AE times in the CTL group. Furthermore, we compared the two correlation coefficients with a Z-test after Fisher Z-transformation and they were not significantly different (*p* = 0.61).

### Pain

Pain levels in people with LLA were low (mean phantom: 6.50 ± 10.6; mean stump: 3.50 ± 9.44), with a maximum reported of 30, either phantom or at the stump level. We checked whether the participants presenting the most pain had significantly different response profiles from the others, but this was not the case.

## Discussion

In this study we investigated the effects of body position and movement’s AE on the temporal characteristics of MI in people with LLA. Specifically, we compared the temporal characteristics of MI performed in congruent and incongruent positions and before and after AE through two locomotor-related tasks (walking 7 m and TUG) in people with LLA and health controls. The main findings are: (1) lower MI times in both groups when imagining walking in a congruent-standing position rather than an incongruent-sitting one, and after actual walking rather than before; (2) no effects of body position and order of AE on MI times for the TUG task in either group; and (3) different MI’s temporal accuracy, with an overestimation of AE times during MI in the LLA group and an underestimation in the CTL group.

When considering the MI times of walking, the *Position* effect was similar in both groups, with 5.5% shorter MI times on average when MI was performed in a congruent-standing position compared to an incongruent-supine position. This finding is consistent with those from numerous studies reporting that the actual position of the body parts influences the MI of upper-limb movements^21–30^. Namely, these studies repeatedly show shorter MI times when participants’ upper-limb positions are congruent or compatible compared to incongruent or incompatible with the movements. This finding is also in line with that of a study in which healthy younger and older adults were faster to imagine walking in a standing rather than a sitting position^31^. Hence, the present results extend previous findings and show that the actual body position may actively interfere with the MI of walking in both healthy individuals and people with LLA. As proposed by Saimpont et al.^31^, the MI of walking may be facilitated when individuals are ready for walking (standing) and hindered when they lie in a supine position. Actual proprioceptive information—either congruent or incongruent—from multiple body parts may influence the MI of walking. When this information is congruent, it could be easier or faster to access and process, and thus MI times would decrease. In line with this assumption, Mercier et al.^28^ showed that MI with eyes closed of finger movements, performed either in a compatible or incompatible hand position, was not facilitated in the compatible position in a deafferented subject, whereas it was so in the CTL group. One limit of this interpretation, however, comes from the absence of control for the ease or difficulty to assess the 7 m distance from a standing position compared to a supine one. The assessment of walking distance may be more difficult from the supine position, and whether this factor is confounding in MI times should be further investigated.

During the walking task, the *Order* effect elicited similar response patterns in both groups. Indeed, MI times were10.6% shorter, on average, when walking was imagined after AE rather than before. These results extend those of previous studies showing that the variability of MI times is significantly reduced when the time elapsed from AE decreases^19^ or when MI trials are alternately carried out before or after AE trials rather than before^32^. In these studies, MI times per se did not change, but less variability in MI times can be considered as reflecting more ease in imagining movements. A crucial difference between executed and imagined movements is that no updated bottom-up information is available during MI, which suggests that, when MI is performed before AE, the sensory information associated with the ongoing movement has to be retrieved from long-term procedural memory. Conversely, when MI is performed after AE, this information may be retrieved in short-term memory, which would, in turn, shorten the generation and manipulation of the mental images and body sensations. Given our experimental design, we cannot rule out that MI performed after AE might have benefited from MI performed before AE. Thus, the *Order* effect might be due to either or both previous AE and MI trials.

The fact that the *Position* and *Order* effects influenced the MI times of walking in the same way in the LLA and CTL groups might be explained by the relative influence of the missing segment in such a complex functional task. As walking requires the coordination of movements of the lower and upper limbs, it is possible that the congruent proprioceptive information from several other body parts (*Position* effect) and the sensory information stored in short-term memory after AE (*Order* effect) were sufficient to provide the facilitation effects. The Bayes factor for the *Group* effect, the *Position* × *Order* interaction and the *Position* × *Order* × *Group* interaction were between 1/3 and 3, indicating that these non-significant results were non-evidential (see Methods). More data are thus needed to investigate these effects and interactions.

During the TUG task, the body position and AE did not influence MI times in either group (on average less than 3.5% of time difference between conditions), as emphasised by the Bayes factors under 1/3 for the main effects of *Position* and *Order*, as well as for the interactions. This could be mainly due to the task characteristics of the TUG, which requires changes in positions. Indeed, the sitting position characterised here as congruent was actually congruent with only a small part (the beginning) of the TUG task since most movements (walking and turning around) were performed in an erected position during this sequence. Moreover, since the TUG task involves a variety of subtasks (i.e. standing, walking, turning and sitting down), this variety makes it harder to imagine them. Hence, it took twice as long to complete them than the walking task in both groups, which could have masked underlying facilitation effects of *Position* and *Order*.

Participants with LLA took more time than healthy controls to physically perform both the walking and TUG tasks. This was expected, as they were tested at the beginning of the locomotor prosthetic training (mean time since prosthesis wearing was 27.2 ± 15.9 days, and mean prosthesis wearing time was 5.1 ± 3.3 h per day). Considering the MI/AE time ratios, whatever the task and condition of MI, they were higher in the CTL group than in the LLA group. Furthermore, ratios were higher than 1 in the CTL group and lower than 1 in the LLA group. Healthy controls thus overestimated AE times during MI. Although similar MI and AE times have generally been shown for cyclical movements such as walking^19,32,33^, MI times may be longer than AE times when participants are instructed to form vivid images and sensations of movements without time constraints^31,34^. For their parts, individuals with LLA underestimated AE times during MI despite receiving the same instructions as the controls, which may be due to the slight experience of walking with a prosthesis and the fact that they referred to their walking capacities or speed prior to amputation when imagining walking. Indeed, Saruco et al.^16^ previously reported that MI times were closer to AE times after locomotor training in people with LLA. A similar explanation may also account for the TUG task, even though MI times tended to be longer in the LLA group than in the CTL group, possibly because of the greater perceived difficulty of this task^34^. Since AE times in both groups were consistent throughout the testing session, we are confident that the changes in the ratios were related to changes in MI times.

Concerning the walking task, it is important to point out that the MI/AE time ratios were close to 1 in the congruent-standing position and after AE in the CTL group, but they moved away from 1 in the LLA group. Assuming that 1 reflects a perfect isochrony between MI and AE, the reduction of MI times during the congruent-standing position and after AE improved MI’s temporal accuracy in the CTL group but not in the LLA group. These findings underline the importance of analysing MI times as such, as well as MI/AE time ratios, when investigating the temporal characteristics of MI and that faster MI does not necessarily mean better MI. As stated in the introduction, MI would rely on motor predictions performed by internal models^19,20^. As these models partly base their predictions on the actual body information, the MI of walking performed in a congruent-standing position could have improved these predictions. Similarly, after AE, the internal models could have been refined by the physical trials, which updated sensory information by providing feedback so that it shortened the time to imagine walking. However, these potential explanations would only hold for healthy individuals since motor predictions were worse in people with LLA in the congruent-standing position and after AE, suggesting that the internal models were not accurately updated in this group.

Participants performed only two or three MI trials by condition in the present study, which could explain the lack of internal models updating in the LLA group, especially considering that they were at the beginning of their prosthetic use. In line with this assumption, Saruco et al.^16^ (2019) show that MI’s temporal accuracy significantly improves in people with LLA after weeks of prosthetic use. Interestingly, older adults showed an increase in MI’s temporal accuracy even within a single training session of 20 MI trials^35^. It would thus be interesting to test whether MI’s temporal accuracy would improve in people with LLA after several MI repetitions of walking within a single session and whether this process would be enhanced by performing MI in a congruent-standing position and after AE. In the same way, we can wonder whether the decrease in MI times would stop in healthy individuals if MI times matched AE times after several repetitions.

The Bayes factor for almost all interactions in the walking task and all interactions and simple effects in the TUG task were close to 1, indicating that these non-significant results were non-evidential. Hence, more data are needed to investigate these effects and interactions.

Finally, we tested whether individuals within each group showed the same pattern for both functional tasks. The significant correlations between the MI/AE time ratios for the walking and TUG task for each group confirmed an inter-task consistency which suggests robust individual patterns of MI within each group in this single testing session, regardless of the task duration and complexity—two factors that could have led to changes in response patterns^34^. The present results also indicate that the consistency in MI’s temporal accuracy for locomotor tasks could be an individual trait not altered after LLA. Such consistency is likely due to the main features of the two tasks, which are based on locomotion. In line with this hypothesis, changes in MI’s temporal accuracy in individuals with LLA after several sessions of MI were similar for a walking task and a TUG task^16^.

Although this study reports original findings, the sample was small. Furthermore, we included only three people whose amputation was due to peripheral vascular disease and four people older than 65, whereas most LLAs have a vascular origin and mainly affect people above 65. Thus, one should be careful in generalising these results. Further studies should include larger samples of people with LLA, with characteristics closer to those usually found in this population.

In sum, in both healthy controls and people with LLA, it took less time to imagine walking in a congruent-standing than in an incongruent-supine position and after AE compared to before AE. Concerning the MI’s temporal accuracy, the CTL group overestimated AE times during MI, whereas the LLA group showed an underestimation. Additionally, shorter MI times in the congruent-standing position and after AE led to better MI’s temporal accuracy in the CTL group, whereas the opposite occurred in the LLA group. A congruent body position and AE facilitated information processing, with easier or faster access to the sensory information needed for MI. Refinement of internal models’ accuracy and motor predictions would only occur after several training sessions, at least for people with LLA. Whether these short-term effects of body position and AE may facilitate internal models’ long-term refinement in people with LLA remain to be tested. These results may have practical implications for the use of MI training to rehabilitate people with LLA. First, these individuals should adopt a body position as congruent as possible with the starting position of the imagined movements. Second, as already proposed by Malouin et al.^9,11^, physically performing the task before working on its mental representation is crucial to prime and facilitate MI.

## Methods

### Participants and design

Ten participants with LLA (mean age = 59.6 ± 13.9 years, four females) and ten gender- and age-matched healthy control participants (mean age = 60.1 ± 15.4 years, four females) were included in this study. Participants with LLA were recruited from the Amputation Program at the Institut de Réadaptation en Déficience Physique de Québec (IRDPQ, Quebec City) and were the same as those investigated by Saruco et al.^16^, whereas controls were recruited from the university campus and the rehabilitation centre.

Inclusion criteria for participants with LLA were: (1) aged between 18 and 85 years; (2) a unilateral LLA (transtibial or transfemoral); (3) enrolled in a locomotor prosthetic training program; and (4) able to wear their prosthesis during at least 1 h per day and walk with it for at least 10 min. Exclusion criteria were: (1) already trained with a prosthesis for this amputation; (2) a chronic illness such as cancer or dementia; (3) a neurologic condition such as stroke or a traumatic brain injury; (4) a score lower than 24/30 on the Mini-Mental Status Examination^36^; and (5) an inability to perform MI as assessed with the Time-Dependent Motor Imagery Screening test^37^. The experiment was conducted in accordance with the Declaration of Helsinki, and the protocol was approved by the ethical committee of the IRDPQ (#2012-287). Informed consent was obtained from all participants.

### Experimental procedures

To prevent fatigue, participants of the LLA group participated at the same time in two sessions separated by one day. In the first testing session, they were administered an adapted version of the Kinesthetic and Visual Imagery Questionnaire (KVIQ,^38^) to assess MI vividness and control that they were able to form images and sensations of movements (see Saruco et al.^16^ for the procedure and complete results). In the second session, the participants assessed their actual pain and then performed two chronometric tests to investigate MI and AE times. Participants in the CTL group were administered the KVIQ and performed the chronometric tests in a single session. The same experimenter (A. D.) conducted all tests.

#### Assessment of pain

Chronic or high levels of phantom and stump pain may alter MI accuracy^39,40^. The mean intensity of phantom limb and stump pain in the participants with LLA was thus assessed before the chronometric tests by means of a verbal analogue scale ranging from 0 (no pain at all) to 100 (worst pain possible).

#### Assessment of the temporal characteristics of MI

**Figure 4:**
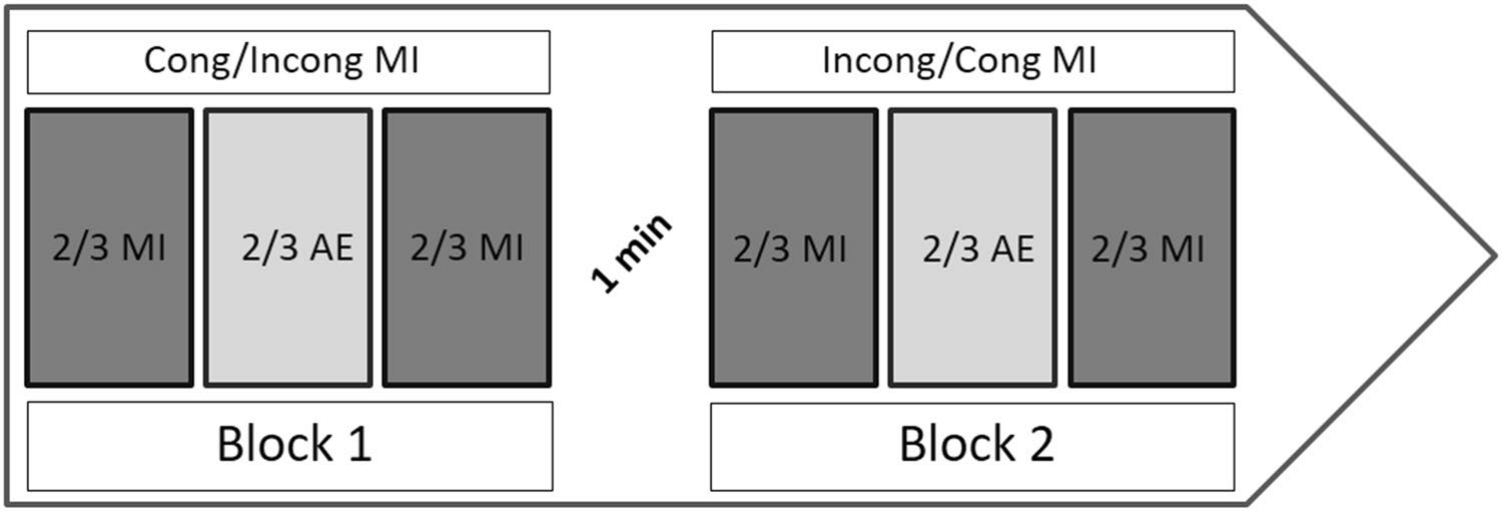
Time course of the MI and AE trials, for each task. Participants performed two blocks of trials, each being composed of two or three MI trials (if the time difference between the first and the second trial was higher than 10%, a third trial was carried out), followed by two or three AE trials, followed again by two or three MI trials. During the first block, participants had to imagine the task in one of two body positions, either congruent (Cong) or incongruent (Incong) with the action. During the second block, they had to imagine the task while adopting the other body position. The order of the blocks was counterbalanced across participants, and a 1 min rest period separated the two blocks.

We assessed MI and AE times for two locomotor-related tasks (see Fig. 4 and below for details). For each task, after a demonstration by the experimenter, participants performed two blocks of trials, each being composed of two or three MI trials (if the time difference between the first and the second trial was higher than 10%, a third trial was carried out), followed by two or three AE trials, followed again by two or three MI trials. During the first block, participants had to imagine the task from one of two body positions, either congruent or incongruent with the action. During the second block, they had to imagine the task while adopting the other body position. The order of the blocks was counterbalanced across participants, and a 1 min rest period separated the two blocks.

During MI, participants had to close their eyes and imagine the task at a self-paced speed, as vividly as possible, from a first-person perspective, by mentally seeing and feeling their limbs in action and without any actual movements. The experimenter recorded the time for each trial (either AE or MI) with an electronic timer; the recording started with an auditory signal given by the experimenter and stopped when the participant verbally signalled the end of a trial. Participants used a walking aid if needed for security purposes. In such cases, they also imagined performing the task with the aid. To check for MI compliance, participants self-rated MI vividness after each imagined trial with the same 5-point analogue scale used in the KVIQ.

##### Walking task

First, participants performed a simple walking task on a 7 m walkway delimited on the floor with a start and a finish line using tape. They had to imagine walking from (1) a standing (congruent) position in front of the start line and (2) a supine (incongruent) position on a treatment table placed next to the start line until they reached the finish line. In the supine position, participants first imagined themselves standing and verbally signalled to the experimenter the moment when they were mentally upright. The experimenter then gave the starting signal.

##### Timed-up-and-go task

Second, participants performed the TUG task, which consisted of standing from a sitting position in a chair, walking 3 m until a line delimited on the floor using tape, turning around, walking back to the chair and sitting down. Participants imagined the TUG from (1) a sitting (congruent) position on a chair placed in front of the start line and (2) a supine (incongruent) position on a treatment table placed next to the start line. From the supine position, they first imagined themselves sitting and verbally signalled when they were mentally seated before the experimenter gave the starting signal.

### Data and statistical analysis

Two participants in the LLA group (participants 4 and 5) could not perform MI in the supine position due to back pain. We thus processed data for eight amputees and 10 controls. We calculated MI times for each participant and condition by averaging the data of the two or three trials. In the same way, we calculated AE times for each participant and condition. We used the MI/AE time ratio as an indicator of MI’s temporal accuracy. We calculated ratios for each trial and averaged them for each condition. We analysed AE times, MI times, and MI/AE time ratios separately. We routinely analysed data for normality distribution using the Shapiro–Wilk test. When the assumption of normality was not met, we performed an aligned rank transform (ART) for non-parametric factorial analysis^41^. Unlike common non-parametric tests, such as the Friedman test, the ART procedure allows examining interaction effects by aligning data, applying averaged ranks and conducting separate analyses of variance (ANOVAs) on the aligned ranks data for each main effect and interaction (see Wobbrock et al.^41^ for details). Finally, we performed correlation analyses with the Spearman test to explore the relationship between the TUG and walking MI/AE time ratios in the two groups. We interpreted all effects with the *p*-values while also reporting the Bayes factors for non-significant results. *B*_HN(0, x)_ referred to a Bayes factor in which the predictions of H1 were modelled as a half-normal distribution with a mode of 0 and an SD of *x*, where *x* scales the rough size of the effect that could be expected. Since previous research lacked SDs, we used the room-to-move heuristic to estimate them^42^ by using one condition or group to define the maximum difference (i.e. the size of the room) that could be obtained between conditions or groups. Then, the SD of the half-normal distribution was set as a maximum divided by 2 (see Dienes^42^ for details). We considered a Bayes less than 1/3 as suitable enough evidence for H0 over H1 and a Bayes factor between 1/3 and 3 as non-evidential.

#### Walking task

Actual execution times and MI times were normally distributed in each group and condition, thus allowing a mixed ANOVA with factors *Group* (LLA or CTL) and *Position* (Congruent-Standing or Incongruent-Supine) to compare AE times and with factors *Group* (LLA or CTL), *Position* (Congruent-Standing or Incongruent-Supine) and *Order* (MI performed before AE or After AE) to compare MI times. Concerning AE times, the *Position* factor corresponds to AE performed when participants either imagined the movements in the congruent-standing position or in the incongruent-supine one, but AE was always performed in the right position. MI/AE time ratios were not normally distributed in each group and condition. We thus conducted an ART procedure followed by mixed ANOVAs with factors *Group* (LLA or CTL), *Position* (Congruent-Standing or Incongruent-Supine) and *Order* (Before AE or After AE).

#### Timed-up-and-go task

Actual execution times and MI times were normally distributed in each group and condition. We thus performed a mixed ANOVA with factors *Group* (LLA/CTL) and *Position* (Congruent-Sitting/Incongruent-Supine) to compare AE times, and with factors *Group* (LLA or CTL), *Position* (Congruent-Sitting or Incongruent-Supine) and *Order* (Before AE or After AE) to compare MI times. The same remark as for the walking task for the denomination of the *Position* factor for AE times applies here. MI/AE time ratios were normally distributed in each group and condition, thus allowing an ANOVA with factors *Group* (LLA or CTL), *Position* (Congruent-Sitting or Incongruent-Supine) and *Order* (Before AE or After AE) for comparisons.

## Data Availability

The datasets analysed during the current study are available from the corresponding author on reasonable request.
